# Respiratory internal kinematics of the tongue base and soft palate in obese minipigs with obstructive sleep apnea

**DOI:** 10.21203/rs.3.rs-3581408/v1

**Published:** 2023-11-16

**Authors:** Zi-Jun Liu, Mandy Yang, Meng-Zhao Deng, Mohamed Yehia Abdelfattah, Michael Baldwin, Edward Weaver

**Affiliations:** University of Washington; Universiyt of Washington; Universiyt of Washington; University of Washington; University of Washington; University of Washington

**Keywords:** Tongue base, soft palate, respiration, obesity, obstructive sleep apnea

## Abstract

It is largely unknown how the tongue base and soft palate deform to alter the configuration of the oropharyngeal airway during respiration. This study is to address this important gap. After live sleep monitoring of 5 Yucatan and 2 Panepinto minipigs to verify obstructive sleep apnea (OSA), 8 and 4 ultrasonic crystals were implanted into the tongue base and soft palate to circumscribe a cubic and square region, respectively. The 3D and 2D dimensional changes of the circumscribed regions were measured simultaneously with electromyographic activity (EMG) of the oropharyngeal muscles during spontaneous respiration under sedated sleep. The results indicated that both obese Yucatan and Panepinto minipigs presented spontaneous OSA, but not in 3 non-obese Yucatan minipigs. During inspiration, the tongue base showed elongation in both dorsal and ventral regions but thinning and thickening in the anterior and posterior regions respectively. The widths showed opposite directions, widening in the dorsal but narrowing in the ventral regions. The soft palate expanded in both length and width. Compared to normal controls, obese/OSA ones showed similar directions of dimensional changes, but the magnitude of change was two times larger in the tongue base and soft palate, and obese/OSA Panepinto minipigs presented 10 times larger changes in all dimensions of both the tongue base and the soft palate. The opposite direction of the respiratory spatial relationship between these two structures was seen in obese/OSA as compared to normal minipigs.

## Introduction

Respiration is a critical component of oropharyngeal function. The tongue base and soft palate are the two key structures of the oropharynx, and their sizes/volumes, shapes, and spatial relationships significantly affect oropharyngeal patency ^1–9^. As a part of the oropharynx, the tongue base impacts the entrances to the larynx for respiration, directs contents to the esophagus for swallowing, and contributes to resonance for vocalization ^10^. The soft palate, as a muscular and aponeurotic fold suspended from the posterior border of the bony hard palate, also serves multiple important oropharyngeal functions. Its aponeurosis provides a ballast against which the tongue base propels food from the oral cavity to the pharynx. The palatal muscles pull the soft palate posteriorly and superiorly to close the velopharynx during swallowing or certain phonations. Thus the soft palate also plays an important role to regulate the airflow through nose and/or mouth ^11^. Clinical studies have demonstrated that in obstructive sleep apnea (OSA), respiratory disturbances are more common at the soft palate ^12^ but more severe at the tongue base ^13^. Therefore, the internal kinematics of these two structures play a critical role in maintaining or compromising the oropharyngeal airway. However, how these two key structures alter their shapes and spatial relationship to configure the oropharyngeal airway during respiration is largely unknown, especially during sleep. The present study was to address this important gap by using implantable ultrasonic crystals to quantify the respiratory 3D and 2D dimensional changes in the tongue base and soft palate respectively during sleep in normal non-OSA and obese OSA minipig models.

## Materials and Methods

### Animals and OSA verification

Three normal (2 females and 1 male) and 2 obese (1 male and 1 female) Yucatan (aged 8–11 months, Premier BioSource, CA), and 2 obese Panepinto (males, aged 6.5 years, donated by Panepinto & Associate, Mason Ville, CO) minipigs were included in the present study. Panepinto minipig is a crossbreeding between Yucatan and Vietnamese minipig ^14^. The ranges of body weights and the body mass index (BMI) were as follows: normal: 46 to 55Kg with the BMI of 38 to 39 Kg/m^2^; obese Yucatan: 68 to 71Kg with the BMI of 50 to 51Kg/m^2^; and obese Panepinto: 86 to 104Kg with the BMI of 49 to 59 Kg/m^2^. All procedures were approved by the Institutional Animal Care and Use Committee of the University of Washington (Protocol# 3393–04).

Before the terminal procedures for the internal kinematics of the tongue base and soft palate, OSA was verified and characterized on all 7 minipigs during sedated and spontaneous sleep. The results of the wireless and remote sleep polysomnography and airflow dynamics were published previously ^15,16^. In summary, OSA was verified in all 4 obese minipigs with the apnea-hypopnea index (AHI) ranging from 30 to 59, and the AHI in 3 normal minipigs were 0 to 5. This verification also demonstrated the similarity of respiration parameters during sedated and spontaneous sleep, indicating the sedated sleep can be a surrogate of the spontaneous sleep.

### Terminal procedures

**Figure 1:**
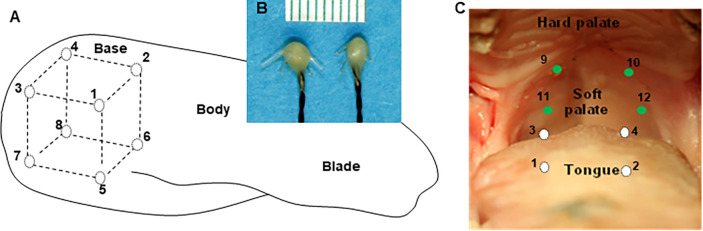
Legend not included with this version.

Under anesthesia, the pig was placed supine. A submandibular incision was first made, and subcutaneous tissues were separated to expose the bilateral genioglossus (GG), styloglossus (SG), and thyrohyoideus (TH). A pair of 0.10mm nickel–chromium wire electrodes (California Fine Wire, Grover Beach, CA) with 1mm bared tip and 2mm separation between the wires were inserted into each of these 3 muscles along their fiber direction using a 25G needle. Then, the two mouth openers were inserted into the bilateral molar region to keep the gape at 35–40 mm, and the tongue was pulled forward to expose the two circumvallate papillae as the border of the tongue base from the tongue body. Eight 2mm B barbed ultrasound crystals (Sonometrics Co. London, Canada) were implanted into the tongue base through the submandibular approach. During implantation, a small tunnel was first made by a dull long-beaked straight hemostat, then a barbed crystal was inserted using another long-beaked straight hemostat. The location of each crystal was confirmed by transoral palpation of the tongue base by the fingers of the operator. Therefore, the dorsal surface of the tongue was kept intact without penetration. The implanted crystals in the tongue base were secured by their barbs and leading wires were further sutured to the nearby facial tissues. As shown in Fig. 1A, these 8 crystals circumscribed a cubic region in the tongue base: crystals #1 and #2 were implanted 2mm posterior to the two circumvallate papillae and 3mm underneath the dorsal mucosa; the #3 and #4 crystals were 20mm posterior to crystals #1 and #2 respectively and located in the dorsal area. Crystals #5, 6, 7 and 8 were placed in the ventral region at a distance of 20mm from crystals #1, 2, 3 and 4, respectively. In addition, another 4 crystals were directly implanted into the soft palate to circumscribe a rectangular region by using a sharp long-beaked hemostat and were secured in place by their barbs as well (Fig. 1B). The distances between each pair of the ultrasonic crystals in each dimension were consistently 20mm across all animals regardless of their body size and BMI. The leading wires of the crystals implanted into the tongue base were led out from the submandibular incision and the incision was closed in layers by suturing. The leading wires of the crystals placed in the soft palate exited the mouth via the upper retromolar regions of each side.

### Data recording and analysis

After the instrumentation, the pig was situated in the prone position, and the tongue was placed back to its original position. The EMG electrode leads were connected to the MP150 (BioPac. Co. Santa Barbara, CA), and the crystal wires were inserted into the Sonometric input box. The dimensional changes in the tongue base and soft palate along with EMG activity were recorded for 5–10 minutes during respiration using a computer running both Acknowledge (Ver.4.0, BioPac) and SonoLab (Sonometrics) programs. Signals from these two recording systems were synchronized by digital-analog input and output.

Off-line analyses of the crystal signals were performed by selecting 25–30 stable and consecutive respiratory cycles, and the synchronized EMG activity was taken for identifying the inspiratory phase of respiration. Since the initial distances between each implanted crystal pair were consistent (20mm) in all animals (Fig. 1A), the change in each crystal pair during each respiratory cycle was calculated and averaged in each animal. In addition to each selected crystal pair in the tongue base and soft palate, the distances between the crystal pairs of the soft palate and the dorsal tongue base were also sampled and calculated to quantify how the spatial relationships between the dorsal surface of the tongue base and the soft palate changed in respiratory phases.

SPSS (Ver. 19, IBM) for Windows was used for the statistical analysis. Descriptive statistics were calculated for all means, standard deviations (SD), and ranges of dimensional changes. The data were further examined to confirm their normal distribution through skewness calculations. One-way analysis of variance was used to examine the differences among the three types of minipigs followed by Tukey post-hoc tests for pair-wise comparisons. Spearman correlations were calculated to test the associations between the dimensional changes, AHI and BMI. The significant level was set as p < 0.05.

## Results

**Figure 2:**
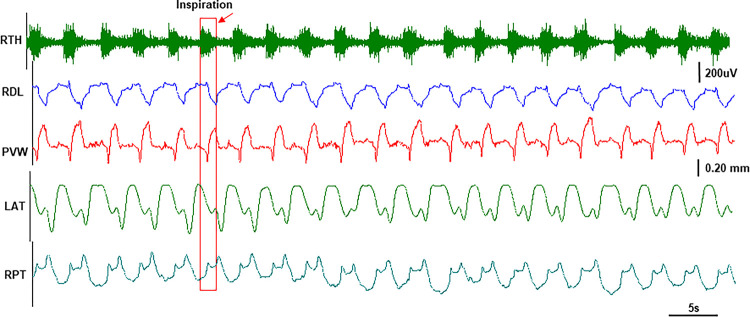
Legend not included with this version.

**Figure 3:**
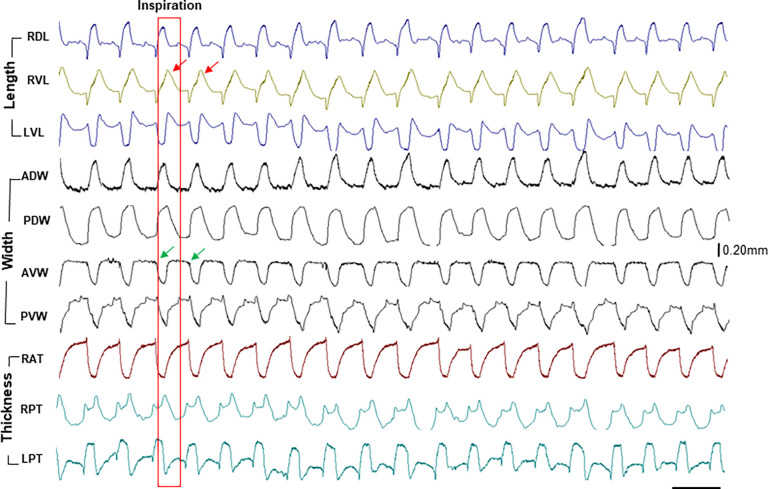
Legend not included with this version.

Corresponding to the respiratory rate, the stereotyped dimentional changes in the lengths, widths, and thicknesses of the tongue base along with the muscle activity bursts were observed (Fig. 2). The respiratory rate was about 16–25 per minute and the ratio of inspiratory/expiratory phases was about 0.65. As marked in Fig. 3, both dorsal and ventral lengths of the tongue base increased during the inspiratory phase. However, the widths of the tongue base increased dorsally and decreased ventrally, indicating widening in the dorsal and narrowing in the ventral tongue base during inspiratory phase. The thicknesses also showed opposite changes in the anterior and posterior tongue base, i.e., became thinner in the anterior and thicker in the posterior tongue base. Therefore, the internal kinematic pattern of the tongue base during inspiration presented the following features: elongation, dorsal widening and ventral narrowing, anterior thinning, and posterior thickening. During expiration, all lengths, widths, and thicknesses presented their dimensional changes in the opposite directions.

**Figure 4:**
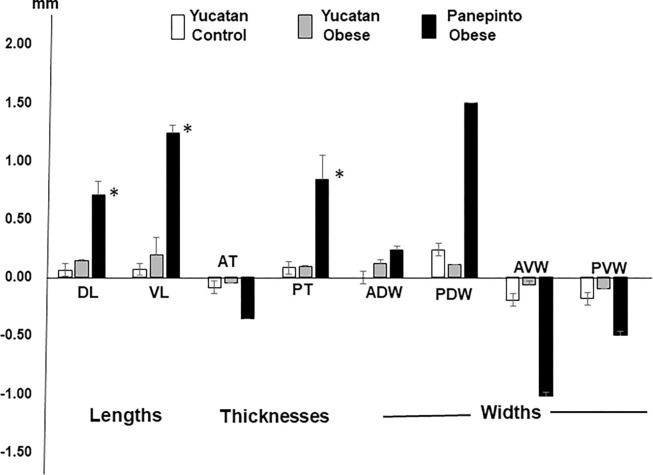
Legend not included with this version.

Since respiration is a symmetric movement and no significant differences were found in the deformational changes in the lengths and thickness between the right and left tongue base as shown in Fig. 3, the values of both sides were averaged to reflect the overall changes in the dorsal/ventral lengths and anterior/posterior thicknesses. These results show that although the patterns of dimensional changes presented similar directions in all minipigs, their deformational ranges were different during respiration. As summarized in Fig. 4, while the normal Yucatan showed the smallest range from 0.05 to 0.23mm, obese/OSA Yucatan minipigs exhibited 2 times larger ranges in elongation (0.14 to 0.19mm) with similar ranges to the normal in the anterior thinning and posterior thickening, and smaller widening and narrowing ranges in the dorsal and ventral parts (0.06 to 0.11mm), respectively. The two aged obese/OSA Panepinto minipigs presented significantly larger ranges with as much as 6–8 times more (0.71 to 1.24, p < 0.05) than the normal group in all lengths, widths, and thicknesses, possibly related to particularly heavy snoring during recording.

**Figure 5:**
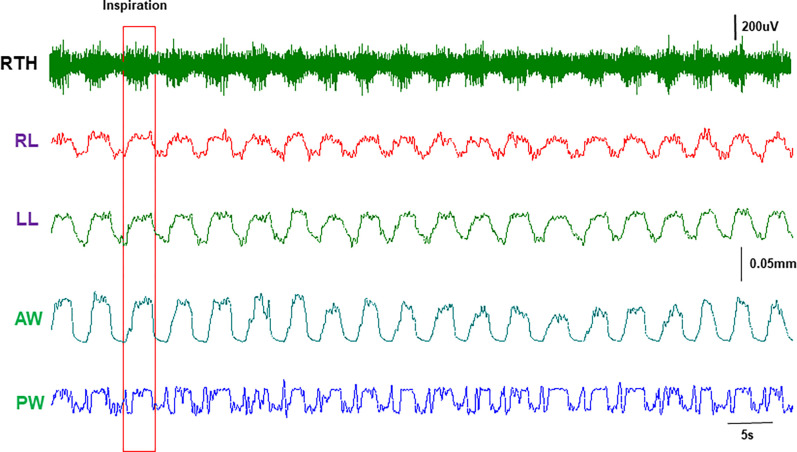
Legend not included with this version.

Due to the difficulty of access and the vulnerability of the crystals, data from the soft palate was limited. Similar stereotyped EMG bursts and dimensional changes of the soft palate were observed (Fig. 5). In the phase of inspiration, the soft palate elongated symmetrically with larger and smaller widening in anterior and posterior regions, respectively in normal Yucatan with the range of 0.02 to 0.05mm. However, the ranges of dimensional changes were more than 2 times larger in obese/OSA Yucatan minipigs (0.09 to 0.17mm), and more than 4 times larger in obese/OSA Panepinto minipigs (0.32 to 0.81mm, p < 0.05).

Although there were no directional alterations of the dimensional changes in the tongue base and soft palate across the three groups of minipigs, the directional alteration of distance between these two oropharyngeal structures was seen during respiration. During inspiration, both anterior and posterior distances between the dorsal tongue base and soft palate were increased (0.09–0.12mm) in normal Yucatan minipigs, indicating the separation of these two key structures. However, the anterior distance of these two key structures in obese/OSA Yucatan and Panepinto/OSA minipigs was shortened (0.42–1.60mm), indicating the possible closure between these two key structures.

The correlation analysis showed that neither AHI, nor BMI was associated with the ranges of dimensional changes during respiration in the tongue base and soft palate.

## Discussion

**Figure 6:**
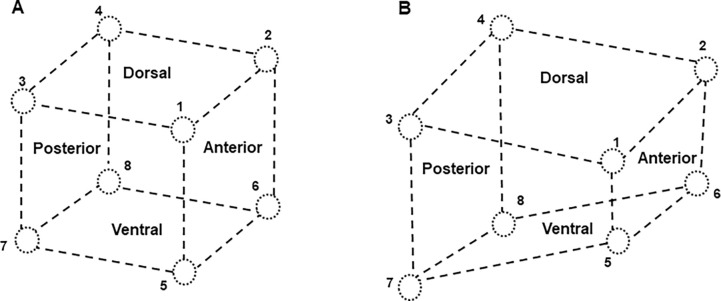
Legend not included with this version.

Both the tongue and soft palate are muscular structures, so the muscular hydrostat theory applies ^17^. However, as explored and published previously, this theory may not be applied to regional dimensional changes of the tongue (and soft plate) as the regional volume changes have been verified ^18^. The present study further demonstrated that the increase and decrease of each measured dimensional change do not compensate for each other to maintain the volume constant in the region circumscribed by the ultrasonic crystals. More importantly, the present study revealed that the elongation and widening of both the tongue base and soft palate may be the key players in leading or guiding the airflow into the oropharyngeal airway during respiration. At the same time, while both the dorsal tongue base and the soft palate widened, the ventral tongue base narrowed instead, along with anterior thinning and posterior thickening of the tongue base. Therefore, during inspiration, the cubic shape circumscribed by the 8 implanted ultrasound crystals (Fig. 6A) becomes an irregular trapezoid-like shape, featuring a longer and wider top but narrowed bottom, and further tapering sagittally from anteriorly decreased to posteriorly increased thicknesses in the tongue base (Fig. 6B). At the same time, the soft palate extends in both length and width. These dynamic dimensional changes in the shapes of the tongue base and soft palate expand the lumen of velar and oral pharynx thus increase the volume of the oropharyngeal airway for its patency, as our findings in the airflow dynamics in the same minipig models previously published ^16^. Thus, it is reasonable to speculate that an enlarged tongue base and/or soft palate due to obesity or other pathological conditions are predisposing factors of airway obstruction, particularly at the status of the decreased tone of the tongue and soft palate muscles during sleep ^19^.

Respiration is a complex process divided into three phases: inspiration, post-inspiration, and active expiration ^20^. During respiration, the morphologies of oropharyngeal structures and the volume of airway spaces continuously change from the anterior nostril to pulmonary alveoli. In addition, the luminal pressure in certain airway segments is proportionally distributed, and the airflow patterns and resistance may therefore be determined by the regional morphological characteristics. A previous study has shown that during oronasal breathing (as during exercise, speech, or smoking), the impedances of the nasal and oral pharynx are determined by the position of the soft palate ^11,21^. Therefore, the observed respiratory dimensional changes in the tongue base and soft palate would certainly alter the morphology of the velar and oral pharyngeal airway which in turn modify the inspiratory and expiratory airflows as we found in the computational fluid dynamic modeling in these same minipig models ^16^.

The present study of the internal kinematics of the tongue base and soft palate in OSA minipig model is particularly relevant to the human OSA condition. A most recent clinical survey indicated that the prevalence of snoring in obese individuals is almost 100% among whom 58% presents severe degree of OSA, and have their airway obstructions, and these obstructions most often occurr at the retro-palatal and retro-glossal levels ^22^. A recent meta-analysis of 2,950 patients from 19 studies also showed the soft palate and tongue base were the two most common sites of airway obstruction. This meta-analysis also showed that the degree of tongue base obstruction was associated with the severity of OSA ^12^.

In the present study, both heavy snoring and severe OSA were identified in obese minipigs although snoring did not occur in young obese Yucatan minipigs during the implanted ultrasound crystal recording. Nevertheless, the enhanced internal kinematics and altered spatial relationship in the tongue base and soft palate during respiration is related to the presence of obesity/OSA, and this enhancement may have a compensatory effect on the potential oropharyngeal airway restriction or collapse.

There are several limitations in the present study. The first is the small sample sizes in each type of minipigs, particularly the lack of the same aged controls of Panepinto minipigs due to the source unavailability. Therefore, the observed differences in Panepinto obese/OSA minipigs could be derived from the different breeds of minipigs. However, given the fact that Panepinto minipigs are a Yucatan crossbreed ^14^, and obese Panepinto had similar BMI to obese Yucatan minipigs, it could be reasonably speculated that the observed differences between normal Yucatan and obese/OSA Panepinto minipigs were most likely resulted from obesity and/or OSA. The second is that not all crystal recordings were successful due to the vulnerability of the implanted ultrasound crystals. Despite these, the results clearly reveal the respiratory characteristics of the internal kinematics in the tongue base and soft palate in normal minipigs and the differences in obese minipigs with OSA. As described in the methods, the recordings were performed under sedated sleep, instead of natural respiration in consciousness or sleep. Fortunately, the confounding effect of sedation and anesthesia on respiration has been proven to be minor ^23–25^, and the physical parameters between sedated and natural sleep present clear similarity in these normal and obese minipigs ^15^. Therefore, this limitation could be considered minor but still needs to be confirmed during natural respiration when the technique becomes available.

## Conclusions

The present study suggests that enhanced respiratory internal kinematics in the tongue base and soft palate are related to the presence of obesity/OSA, and these enhancements may have compensatory effects on the potential oropharyngeal airway restriction or collapse.
